# Mr. Uwins, on Febrifuge Medicines

**Published:** 1800-07

**Authors:** David Uwins

**Affiliations:** Grenville Street, London


### *To the Editors of the Medical and Phyſical Journal.*

GENTLEMEN,

THEORETICAL diſquiſitions and controverſial communications are, perhaps, in ſome meaſure, foreign to the deſign of your publication, as tending to the excluſion of what may be conceived more immediately important and beneficial to the medicinal art: Encouraged, however, by former indulgences, and fully conſcious of the extreme liberality which has hitherto characterized the Medical and Phyſical Journal, I have taken the liberty of again intruding on your notice, by making ſome Obſervations on the *modus operandi of Febrifuge medicines*; or, rather, of ſuggeſting a few hints on this ſubject, and ſubmitting them, with the utmoſt deference, to the Medical Public, through the medium of your uſeful and inſtructive Miſcellany.—To this I am more particularly induced, in conſequence of a miſrepreſentation of a few remarks of mine, inſerted in one of your former numbers. That Dr. HUGGAN ſhould conſtrue my obſervations on his opinions into an oblique imputation of Enthuſiaſm to his encomiums on *opium* in the cure of *febrile diseases*, or a recommendation of *venæsection* in inflammatory fevers, appears altogether unaccountable; as I confidently maintained and aſſerted, on the authority of my own experience, the extreme utility of the former, and the baneful tendency of the latter, in fevers of every deſcription; and I have not the leaſt doubt, that the Doctor's practice in theſe complaints is in exact conformity with my own, though the principle and theory upon which ſuch practice is conducted, may in ſome degree differ.

The ſcience of medicine has, within a few years paſt, received very conſiderable and important improvements, in conſequence of a greater attention having been lately paid to the *causes* than the mere *phenomena* or *symptoms* of diſeaſes; and in no inſtance, perhaps, has this alteration in medical practice been more manifeſt or beneficial, than in the treatment of thoſe complaints which, from their generality and frequency of appearance, pearance, may be confidered as *endemics* in this climate, viz. *fevers.* I humbly conceive, however, that, notwithftanding the improvements which have been already made, and the comparative fimplification which has obtained, we are far from having arrived at a ftate of perfection, either in this or in any other department of the healing art.

A derangement of the nervous fyftem, occafioning *general debility*, is an invariable attendant on fevers of every denomination; and to this fingle caufe, *debility*, are all the fymptoms which occur, under different circumftances of *constitution*, *situation*, *habit*, *&c.* of the patients to be referred: for, notwithftanding the minute divifion and extenfive claffification which have been adopted (and, doubtlefs, with great propriety) by nofological and fyftematic writers on febrile affections, there appears to be no fpecific or abftract difference in the difeafes themfelves, the variety of appearance which they affume being totally dependent upon the ftate of the conftitution receiving the affection. Thus, the fame caufes operating upon a perfon of a fanguine temperament and plethoric habits, fhall occafion the difeafe which has obtained the appellation of *inflammatory fever*, with fymptoms of vafcular excitement; which, on a patient of a contrary defcription, will be productive of a *typhus* or *nervous fever.*

In what fpecific manner the malign powers exert their influence on the fyftem, fo as to occafion that deviation from the healthy ftate, which gives rife to the phenomena of fever; or, in other words, what is the proximate caufe of fever, is not my intention here to enquire; as I conceive it to be a matter of flight confequence to afcertain; the minutiæ of theory being more calculated to difplay ingenuity, and amufe fpeculatifts, than to afford any material information; and an attention to rational principles and conduct in *practice*, is far preferable to thofe *theoretical refinements*, and *fanciful hypothefes*, which are, in the prefent day, by far too prevalent. I do not mean, by this affertion, to give the fmalleft encouragement to that empirical mode of practice, which pretends to preclude the neceffity of all reafoning *a priori* in medicine—far from it. No one, who judges impartially from premifes, will accufe the Poet of advancing any thing in favour of FREE-THINKING, when he afferts, with refpect to modes of faith,

"*His can't be wrong whofe life is in the right.*"

his defign being merely to fhew the fuperiority of *practical piety* over *doctrinal diftinctions*, *theological difputations*, or *religious fanaticifm.*—But, to return, the *counteraction of nervous debility* being the principal objects to which the attention fhould be directed, in the treatment of febrile complaints, the utility utility and abfolute neceffity of proceeding upon a *ftimulant plan* of cure becomes obvious; and thus is the equal benefit derived from the exhibition of *opium* (one of the moft powerful of all nervous ftimuli) in inflammatory, typhus, or any other fever to be explained; this important and truly valuable medicine decreafing the ftrength and number of pulfations, in thofe fevers which are characterized by fymptoms of arterial ftrength and vafcular excitement, and producing effects exactly oppofite, where occafion requires.

That the advantageous effects of opium are to be referred folely to its *ftimulating quality*, is alfo evident from the equal fuccefs attending its adminiftration in the *cold* and *hot* ftages of an intermittent; which is a fact I have fully afcertained from experience.

Nothing can poffibly be more erroneous in theory, or injurious in practice, than the *antiphlogiftic* plan of treatment in febrile diforders; and *depriving* the animal machine of its only fupport, at a time when its functions are impeded, or rendered irregular, folely in confequence of *debility*, or *deficiency* of *excitement*, has been productive of incalculable mifchief!

How then, it may be urged, are we to account for the undeniable benefit which has attended the exhibition of *antimonial preparations*, *nitre*, and other medicines of the *refrigerant* clafs?—A very general mifunderftanding feems to have obtained with refpect to the action of thefe medicines; for a minute examination into their effects, will fhew the propriety of ranking them in the clafs, not of *antiphlogiftics*, or *refrigerants*, (which, by the way, are indefinite terms) but of *ftimulants*. To illuftrate this doctrine, it will be only neceffary to recollect the confequences frequently produced by the adminiftration of antimonials in the incipient ftate of a *nervous fever*. When the quicknefs, fmallnefs, and irregularity of the arterial pulfations, diftreffing pains in the head, extreme oppreffion of the mind, and other fymptoms are prefent, denoting the higheft ftate of *nervous debility*, a dofe of *pulv. antim.* in fuch quantity as to create a flight naufea of the ftomach, will often reduce the pulfe to its proper ftandard, and, by inducing a regularity and due proportion between the action and re-action of the fyftem, will effectually arreft the further progrefs of the difeafe. Can thefe effects be accounted for upon any other principle than a fympathetic excitement of the nervous fyftem, from the temporary derangement in the functions of the ftomach?*

With

* Would not *mercurial oxyds*, or any other mineral preparations, adminiftered with care and attention, be productive of equal advantages with antimonial medicines? and is not the benefit derived from the exhibition of *arfenic. alb.* in fevers, to be referred to its operation on the ftomach?

With respect to *nitre*, its use in *scrophulous* and other diseases, originating in *debility*, and an inactive state of the living powers, as well as the experiments of Dr. *Percival* and others, prove it to possess a stimulating property, which is also further corroborated by the extraordinary success attending its administration in *typhus*, as related by Dr. *Wood*; and a case is somewhere mentioned, of an inflammation of the stomach supervening upon an over dose of this medicine, which is contradictory to the supposition of its acting as a refrigerant. I am moreover informed, that people who are in the habit of taking a large quantity of *acids*, and substances containing much oxygen, are constantly found to have their blood in a *sizy state*.

The above remarks are by no means intended as a recommendation of an indiscriminate and invariable mode of practice in all kinds of fevers; such a doctrine could only be founded in absurdity, and dictated by phrenzy; but are merely for the purpose of endeavouring to simplify the curative indications, and thus to improve the treatment of those diseases, which, on account of their universality, and frequent danger, demand the particular attention of the medical practitioner, and in which an erroneous practice has been productive of more mischievous consequences, and fatal effects, than probably the diseases themselves would have occasioned, had they been left entirely under the guardianship of Nature. I am,

GENTLEMEN,

With the greatest respect,

Your obedient servant,

DAVID UWINS.

*Grenville Street, London,*
*June* 13, 1800.

